# Supplementary material for: Decoding stimulus-specific regulation of promoter activity of p53 target genes
Source: Front Cell Dev Biol. 2025 Jun 13;13:1603603. doi: 10.3389/fcell.2025.1603603 (PMC12202600; doi:10.3389/fcell.2025.1603603)
Supplement: Supplementary file 2 [file DataSheet1.pdf]

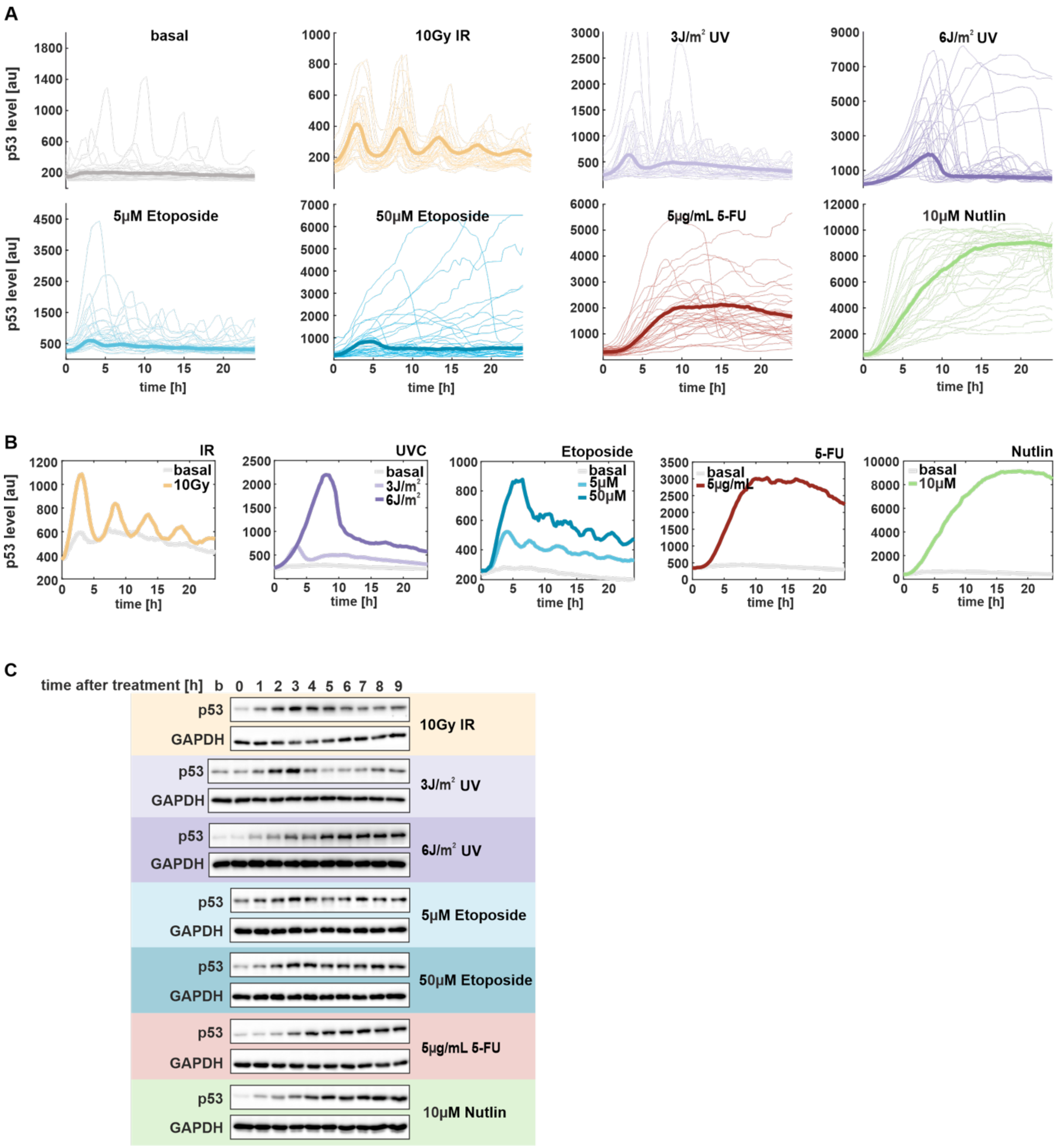

Supplementary Figure 1 – p53 levels were characterized in A549 p53 reporter and wild-type cell lines.

(A) Single-cell trajectories of p53 accumulation in A549 p53 reporter cells in response to the indicated stimuli, measured by live-cell time-lapse microscopy. Representative traces from 30 individual cells are shown. Data is representative of two biological replicates. (**B**) Median trajectories of p53 dynamics in A549 reporter cell line from a replicate time-lapse microscopy experiment. p53 levels are quantified as fluorescent intensity (y-axis) over 24 hours (x-axis). Basal levels are shown in grey. Cells were treated with the indicated stimuli. **(C)** Western Blot measurements of total p53. A549 wt cells were harvested at the indicated time points upon different stimuli. GAPDH was used as loading control. b:basal. Data is representative of three independent repeats.

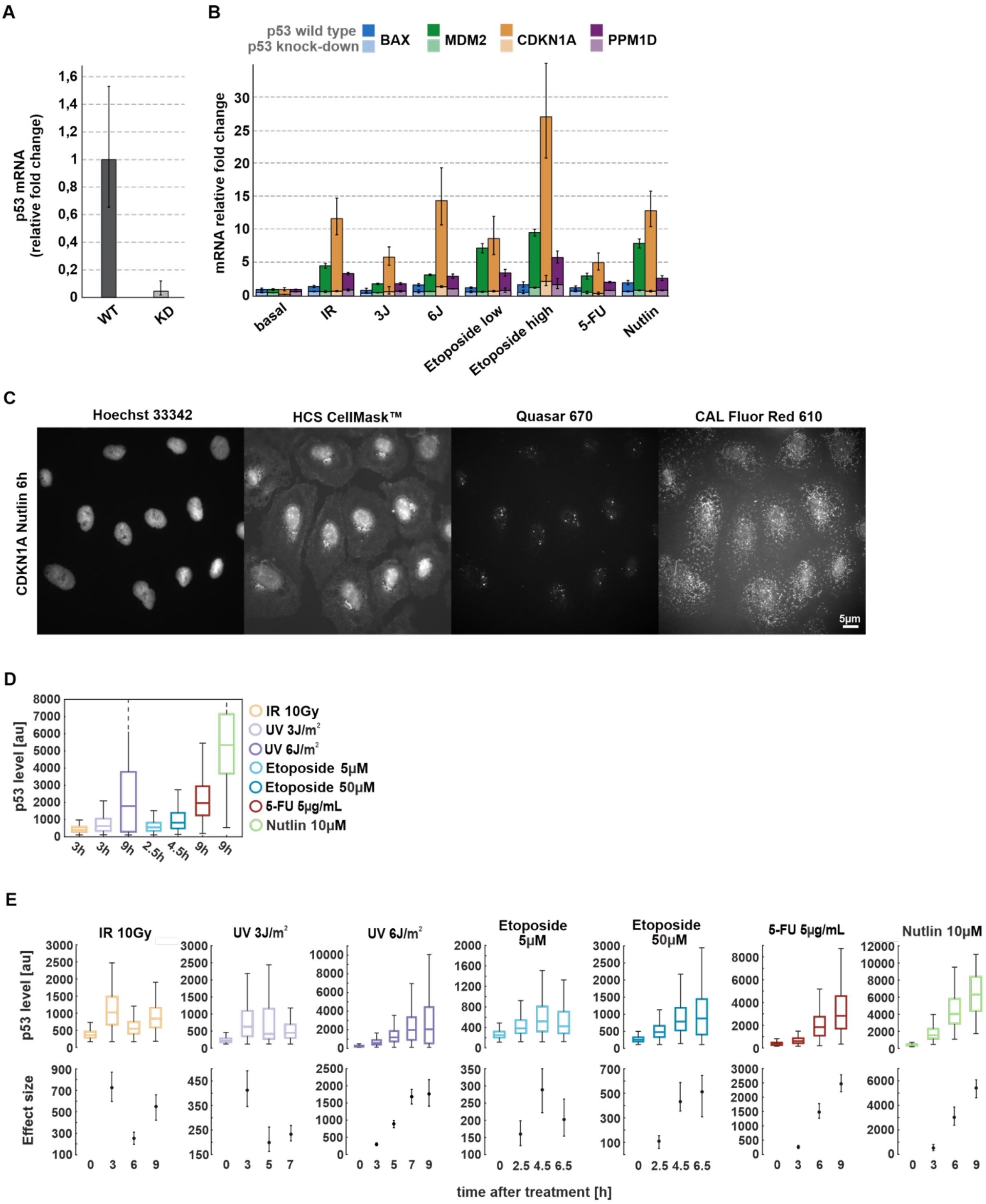

Supplementary Figure 2 – p53 dynamics and target gene expression

(A) mRNA expression of p53 were measured under basal conditions in both A549 p53^{wt} and p53^{sh} cells by RT–qPCR**.** Data represent fold changes relative to the p53^{wt} cell line and are shown as mean and standard deviation from technical triplicates. **(B)** Expression of selected p53 target genes after treatment in A549 wild-type and p53 knockdown cells. mRNA levels were measured by RT–qPCR. The following time points were chosen: 3h post IR, 3J/m^2 UV and etoposide low treatments, 6h after 6J/m^2 UV and etoposide high treatments, 7h after 5-FU and Nutlin addition. Fold changes relative to basal levels are shown for each cell line as mean

and standard deviation from technical triplicates. Data is representative of two or three biological replicates. **(C)** Multicolor fluorescent imaging of exemplary smFISH data. From left to right: individual images of nuclei stained with Hoechst-33342, cytoplasmic staining with HCS CellMask™, intron staining with Quasar 670 dye, and exon staining with Cal Fluor 610. Scale bar corresponds to 5μm distance; images were contrast and brightness enhanced for better visualization. (**D**) Quantification of p53 levels presented as box plots. Time points corresponding to peak p53 accumulation were selected among the ones used in the smFISH experiments to enable comparison across multiple stimuli. (**E**) Replicate measurements of median p53 levels using time lapse microscopy. Data are presented as boxplots, with the bold line indicating medians of distributions and boxes including the 25th and 75th percentiles; whiskers extend to maximum values within 1.5× the interquartile range. Estimated changes relative to basal conditions are shown in the lower plots; error bars represent 90% confidence intervals determined by permutation testing. The number of cells analysed is provided in Supplementary Table 1.

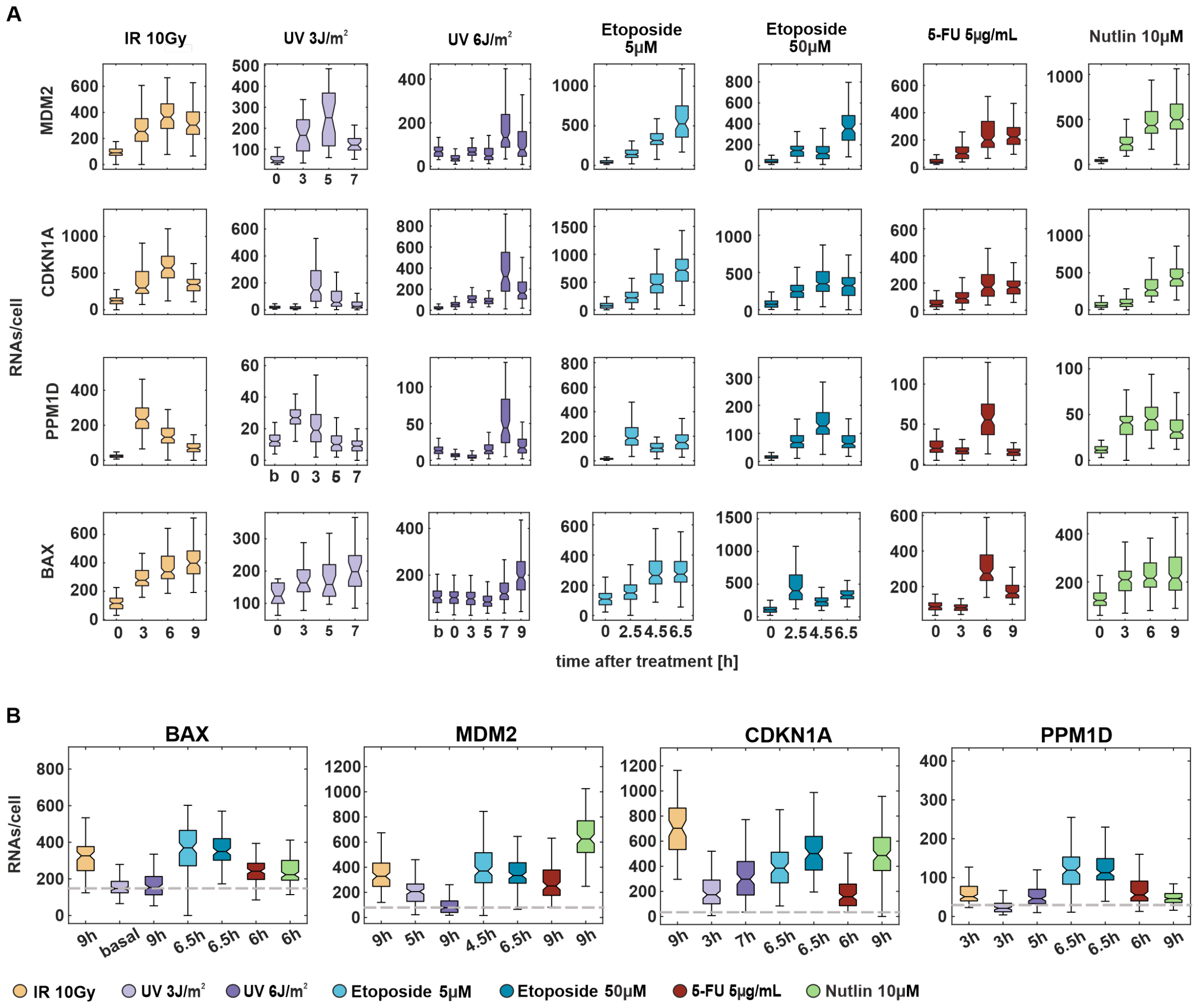

Supplementary Figure 3 – Quantification of mRNA counts across genes and treatments.

(**A**) Replicate measurements of RNAs per cell using FISH-Quant for all target genes and stimuli, displayed as box plots. Lines indicate medians of distributions; boxes include data between the 25th and 75th percentiles; whiskers extend to maximum values within 1.5× the interquartile range. The number of cells analysed is provided in Supplementary Table 1. (**B**) Quantification of RNAs per cell using FISH-Quant for four target genes, presented as box plots. Time points corresponding to peak RNA levels were selected to compare gene expression across different stimuli. Black lines indicate the mean of each distribution, while boxes represent the interquartile range (25th-75th percentiles). Dashed lines denote the mean RNA levels under basal conditions, calculated across all the stimuli for each gene.

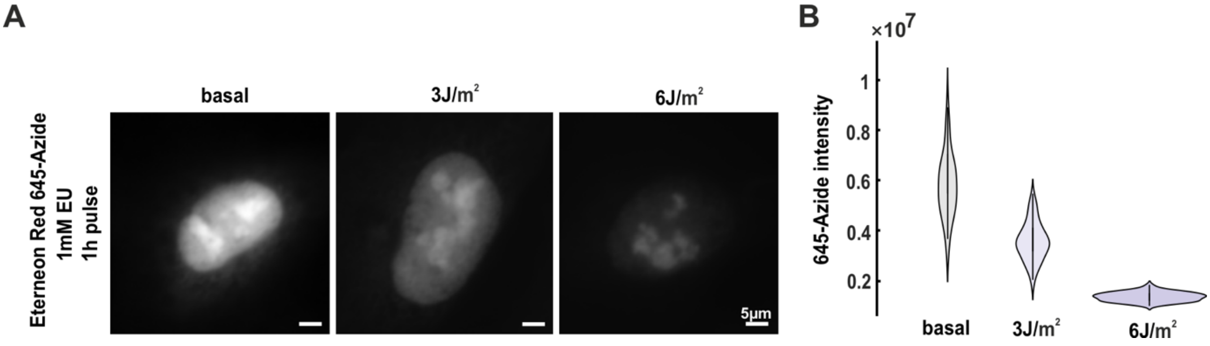

Supplementary Figure 4 - RNA synthesis is globally inhibited upon UV radiation.

(A) Representative images of metabolic labelling of nascent RNAs, showing decreasing of RNA synthesis after indicated doses of UV radiation. A549 wt cells were incubated with 1mM EU for 1h and fixed 3h after treatment. They were subsequently stained with 20µM Eterneon Red 645-Azide and Hoechst-33342. **(B)** Fluorescence intensity was then quantified via ImageJ. Scale bar corresponds to 5µm distance; images were contrast and brightness enhanced for better visualization. Data is representative of two biological repeats.

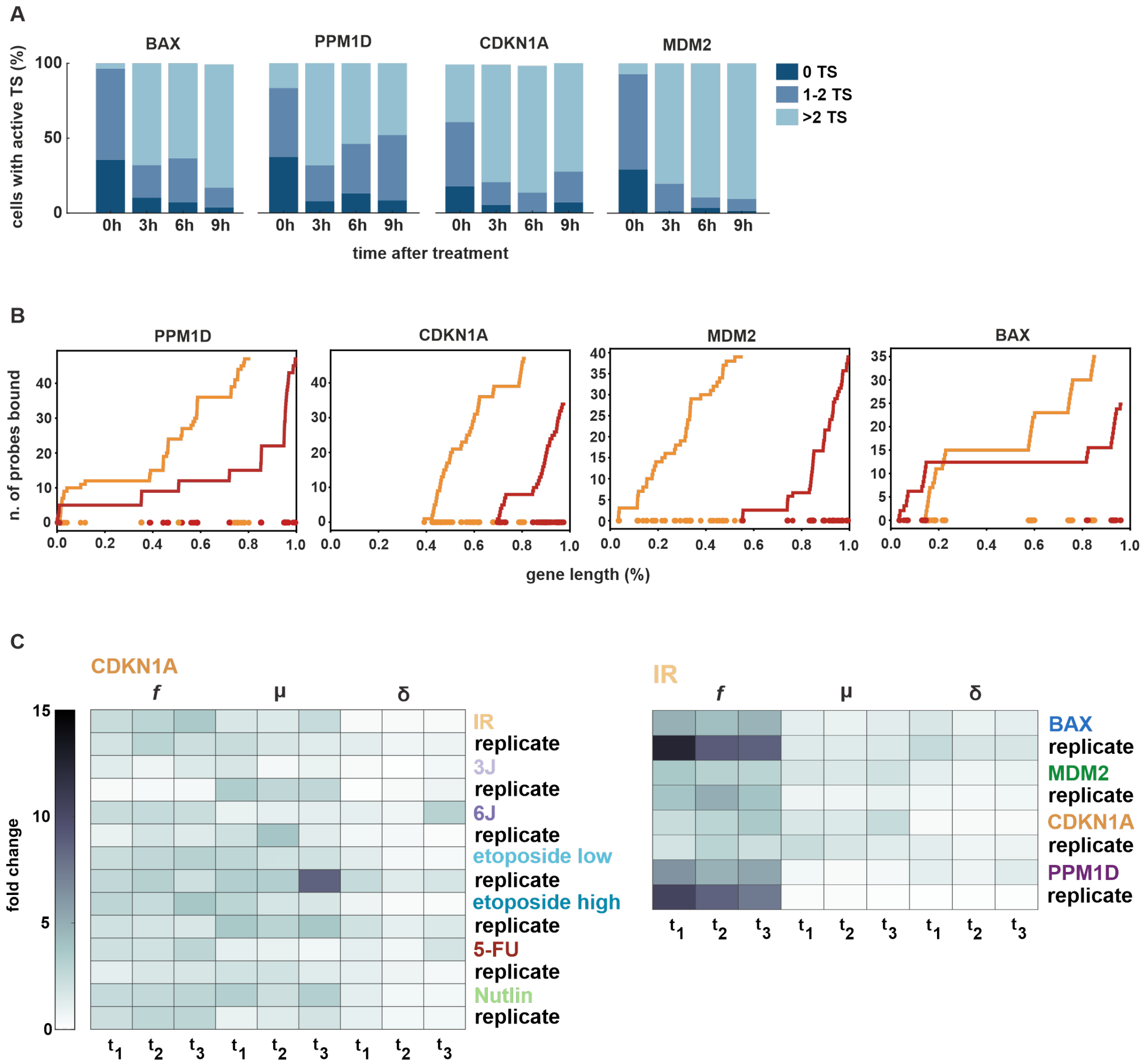

Supplementary Figure 5 - Analysis of promoter activity for p53 target genes.

(A) Stacked bar plots representing the percentage of cells with 0, 1 or more than 2 active transcription sites (TSs). Data are obtained from smFISH measurements after Nutlin treatment at the selected time points. The maximum number of genomic loci was previously validated in Friedrich et al. 2019 as follows: BAX (4), PPM1D (4), CDKN1A (4), and MDM2 (3). **(B)** Plots show the number of intron (in yellow), and exon (in red) probes bound to transcribing Pol II (y-axis) along the genomic sequence of each p53 target gene, shown in percentage (x-axis). Reference sequences (hg38) were used without 3' and 5' UTRs: chr19:48956199-48961097 (BAX), chr12:68808478-68839849 (MDM2), chr6:36684102-36685800 (CDKN1A), chr17:60600415-60663552 (PPM1D). **(C)** MCMC estimates of f, μ, and δ represented in a heatmap. Left panel: Changes in transcriptional parameters at the CDKN1A promoter in response to selected stimuli. Right panel: Changes in transcriptional parameters of p53 target gene promoters following IR. Each column represents a specific time point (3h, 6h and 9h for IR, 5-FU and Nutlin; 2.5h, 4.5h and 6.5h for etoposide low and high; 0h, 5h, 7h, 9h for UV 3J/m2 and 6J/m2). Data were normalized to basal conditions and shown as fold changes, representing biological replicates for each condition.

Data availability

All measurements derived from smFISH experiments as well as additional output from Bayesian inference are publicly available via the institutional repository of Technical University Darmstadt (https://doi.org/10.48328/tudatalib-1704.2). This includes separate plots of the parameters (f, μ, δ) for each gene and condition with corresponding confidence intervals, histogram fits of the active TS exon fluorescence data, histogram fits of the RNA counts, plots of MCMC convergence, posterior distributions of the parameters inference and fitting of TS quantification. Raw image data are available from the corresponding authors upon reasonable request due to their large size. The corresponding analysis code is available through the same link.

Supplementary Methods

The first factor of the likelihood in Bayesian inference

We want to find an expression for

$$L_{1,c}(\theta;\, k,\, m) = \mathbb{P}\left[X = k, Y = m \,|\, \Theta = \theta\right] = \mathbb{P}\left[\sum_{i=1}^{K} X_i = k, \sum_{i=1}^{K} Y_i = m \,\middle|\, \Theta = \theta\right]$$

We define the partial pmf's

$$p_\theta(m) = \mathbb{P}\left[X_i = 1, Y_i = m\right] \qquad \text{and} \qquad q_\theta(m) = \mathbb{P}\left[X_i = 0, Y_i = m\right]$$

Then, remembering that distributions of sums of random variables are represented by convolutions of those of the summands and considering all combinations of K zeros and ones that sum to k, we get

$$\mathbb{P}[X = k, Y = m] = \left[\binom{K}{k} p_\theta^{*k} * q_\theta^{*(K-k)}\right](m)$$

where p_θ^{*k} denotes the k-fold convolution of p_θ with itself. The partial pmf's p_θ and q_θ are determined from Peccoud and Ycart's expression (see main text for reference) for the corresponding generating functions

$$g_p(z) = M\left(\tilde{\lambda}, \tilde{\lambda} + \tilde{\gamma}, \tilde{\mu}(z-1)\right) - g_q(z) \quad \text{and} \quad g_q(z) = \frac{\tilde{\gamma}}{\tilde{\lambda} + \tilde{\gamma}} M\left(\tilde{\lambda}, \tilde{\lambda} + \tilde{\gamma} + 1, \tilde{\mu}(z-1)\right)$$

with the scaled parameters $\tilde{\lambda} = \frac{\lambda}{\delta}$, $\tilde{\gamma} = \frac{\gamma}{\delta}$ and $\tilde{\mu} = \frac{\mu}{\delta}$, and where $M \equiv {}_1F_1$ is Kummer's confluent hypergeometric function. In order to get the coefficients of the Taylor series at expansion point 0, the following integral formula for M is used. It is correct because the condition $b > a > 0$ holds.

$$M(a, b, x) = \frac{\Gamma(b)}{\Gamma(a)\Gamma(b-a)} \int_0^1 e^{xu} u^{a-1} (1-u)^{b-a-1} du \tag{1}$$

Requiring $a = \tilde{\lambda}, b = \tilde{\lambda} + \tilde{\gamma}, x = \tilde{\mu}(z-1)$, we get

$$\begin{aligned}
g_q(z) &= \frac{(b-a)\Gamma(b+1)}{b\Gamma(a)\Gamma(b-a+1)} \int_0^1 e^{\tilde{\mu}zu} e^{-\tilde{\mu}u} u^{a-1} (1-u)^{b-a} du \\
&= \frac{\Gamma(b)}{\Gamma(a)\Gamma(b-a)} \sum_{n=0}^{\infty} \frac{z^n \tilde{\mu}^n}{n!} \int_0^1 e^{-\tilde{\mu}u} u^{n+a-1} (1-u)^{(n+b+1)-(n+a)-1} du \\
&= \frac{\Gamma(b)}{\Gamma(a)\Gamma(b-a)} \sum_{n=0}^{\infty} \frac{z^n \tilde{\mu}^n}{n!} M(n+a, n+b+1, -\tilde{\mu}) \frac{\Gamma(n+a)\Gamma(b-a+1)}{\Gamma(n+b+1)}
\end{aligned}$$

Consequently, after resubstitution of $\tilde{\mu}$, we get

$$q_\theta(m) = \frac{\Gamma(b)\Gamma(m+a)(b-a)}{\Gamma(a)\Gamma(m+b+1)} M\left(m+a, m+b+1, -\frac{\mu}{\delta}\right) \frac{\mu^m}{m!\delta^m}$$

By the analogous representation of $M(\tilde{\lambda}, \tilde{\lambda} + \tilde{\gamma}, \tilde{\mu}(z-1))$ in the expression for $g_p(z)$, we get

$$\begin{aligned}
\frac{m!\Gamma(a)}{\tilde{\mu}^m} p_\theta(m) &= \frac{\Gamma(b)\Gamma(m+a)}{\Gamma(m+b)} M(m+a, m+b, -\tilde{\mu}) - \frac{m!\Gamma(a)}{\tilde{\mu}^m} q_\theta(m) \\
&= \frac{\Gamma(b)}{\Gamma(b-a)} \int_0^1 e^{-\tilde{\mu}u} u^{a+m-1} (1-u)^{b-a-1} du \\
&\quad - \frac{(b-a)\Gamma(b)}{\Gamma(b-a+1)} \int_0^1 e^{-\tilde{\mu}u} u^{a+m-1} (1-u)^{b-a} (1-u) du \\
&= \frac{\Gamma(b)}{\Gamma(b-a)} \int_0^1 e^{-\tilde{\mu}u} u^{a+m-1} (1-u)^{b-a-1} (1-(1-u)) du \\
&= \frac{\Gamma(b)}{\Gamma(b-a)} M\left(m+a+1, m+b+1, -\tilde{\mu}\right) \frac{\Gamma(m+a+1)\Gamma(b-a)}{\Gamma(m+b+1)}
\end{aligned}$$

Hence,

$$p_\theta(m) = \frac{\Gamma(b)\Gamma(m+a+1)}{\Gamma(a)\Gamma(m+b+1)} M\left(m+a+1, m+b+1, -\frac{\mu}{\delta}\right) \frac{\mu^m}{m!\delta^m}$$

The hypergeometric function was evaluated numerically using the integral expression (1).
